# Supplementary material for: Procalcitonin, C-reactive protein, neutrophil gelatinase-associated lipocalin, resistin and the APTT waveform for the early diagnosis of serious bacterial infection and prediction of outcome in critically ill children
Source: PLoS One. 2021 Feb 5;16(2):e0246027. doi: 10.1371/journal.pone.0246027 (PMC7864456; doi:10.1371/journal.pone.0246027)
Supplement: S1 File — (DOCX) [file pone.0246027.s001.docx]

**S 1 Table**

| Diagnosis | Criteria |
| --- | --- |
| Pneumonia | Respiratory symptoms, signs and focal consolidation on radiograph reported by paediatric radiologist. |
| Bacteremia | Identification of a significant bacterial pathogen in blood by using culture or molecular methods |
| Urinary tract infection | Growth of a single bacterial urinary pathogen at ≥ 10^5^ colony-forming units per milliliter in a normally sterile urine sample in the context of clinical signs of systemic involvement. |
| Meningitis | Identification of a bacterial pathogen in CSF by using culture or molecular methods or clinical methods plus a CSF polymorphonuclear leukocytosis in the absence of an alternative etiological diagnosis. |
| Osteomyelitis | Clinical signs and radiologic confirmation or identification of a pathogen in the blood stream |
| Septic arthritis | Isolation of a bacterial pathogen from a joint |

Supporting Statistical Methods

To investigate the secondary objectives, descriptive statistics were calculated summarizing the baseline patient characteristics for each outcome studied (28-day mortality, prolonged ICU stay, length of ICU stay and duration of IPPV). Univariate tests were applied to identify which variables had a statistically significant association with the specified secondary outcome. For comparing continuous variables, a t-test was applied if the variable was normally distributed, otherwise a Mann Whitney U test was used.  For comparing categorical variables, a Pearson’s chi-squared test or Fisher’s exact test was applied.

A logistic regression model was fit to the binary outcome prolonged PICU stay, modelling the probability of the binary outcome as a function of the explanatory variables. For the continuous outcomes, duration of PICU stay and duration of IPPV, a log-linear model was fitted (the residuals from a linear model suggested that the relationship was non-linear). All independent variables that were found to be significant in the descriptive statistics (a p-value of less than 0.05) were considered as potential candidate variables in each model unless the number of missing values was large. It has been assumed that all values that are missing are missing at random and therefore their inclusion will not introduce a bias into the result. In all cases the variables urinary NGAL, plasma NGAL, resistin, PIM and PIM2 were excluded since more than 350 participants had missing values for each.

To choose which of the candidate variables were included in the final models, a stepwise variable selection was undertaken. Starting with an initial model which includes all possible candidate variables, the algorithm successively adds and removes variables from the model to determine the model that provides the best fit. The model fit is determined using Akaike’s Information Criterion (AIC) which includes a penalty term for the number of parameters in the model and ensures that only variables that have a substantial effect on the performance of the model are included. Note that participants with missing values for any of the variables included in the statistical model had to be entirely excluded.

The model-estimated effect sizes are accompanied by confidence intervals (CIs) which quantify the uncertainty in the estimates. For the logistic regression, the effects are presented as odds ratios which describe the relative difference in the odds of a prolonged ICU stay (vs. a non-prolonged ICU stay) for a given variable. A ROC was also constructed for this model together with the AUC and optimal cut-off point with sensitivity, specificity, NPV and PPV reported. For the log-linear model, the estimates have been transformed by taking the exponential. It is important to note that due to the log-transformation, the effect is no longer additive, but multiplicative. To assess the performance of these models the coefficient of determination (or R^2^) was calculated, which is the percentage of the total variation of the outcome that is explained by the model.

To further investigate the prognostic value of the biomarkers, they were used as potential candidate variables (with no other clinical variables) in a model of prolonged PICU stay. The initial approach was to include the longitudinal blood biomarker data in the model using a generalized mixed-effects model, however, none of the candidate variables were found to be significant. Alternatively, a logistic regression model was constructed using the first recorded values and maximum values of the biomarkers using the same stepwise approach as described above. Transforming the blood biomarker variables by taking the logarithm to base 2 improved the overall fit of the model (the interpretation as the odds ratio is associated with doubling the predictor). The effect sizes and the same performance measures were all calculated as those described above.

**S 2 Table Baseline Characteristics of All Participants and Those with and without SBI.**

|  | Serious Bacterial Infection at Admission to PICU | | Total  *N=657* | P value |
| --- | --- | --- | --- | --- |
|  | No  *N=565 (86%)* | Yes  *N=92 (14%)* |  |  |
| Median Age (years)  IQR | 0.85  (0.27-4.70) | 2.57  (0.81-7.92) | 1.01 (0.3-5.01) | <0.001 |
| Gender- male n (%) | 305 (54.0) | 54 (58.7) | 359 (54.6) | 0.46 |
| Type of Admission, n (%)  -Planned  -Planned following surgery  -Unplanned (emergency)  -Unplanned following surgery | 3(0.5)  375 (66.4)  173(30.6)  14(2.5) | 1(1.1)  2(2.2)  81 (88.0)  8 (8.7) | 4 (0.6)  377 (57.4)  254 (38.7)  22 (3.3) | <0.001 |
| Route of admission, n (%)  -Alder Hey Emergency Department A & E  -Alder Hey High Dependency Unit  -Alder Hey inpatient ward  -Alder Hey theatre  -Other hospital  -Missing | 17 (2.9)  5 (0.8)  84 (14.2)  244 (41.3)  241 (40.8)  1 | 13 (14.1)  0  7 (7.6)  0  72 (78.3)  0 | 30 (4.6)  5 (0.8)  91 (13.9)  244 (37.1)  286 (43.6)  1 | <0.001 |
| Surgery^+^, n (%)  Yes*  -Cardiac  -Neurosurgery  -General  -Orthopaedic  -Thoracic  -Abdominal  -Burns/Plastics  -ENT  -Other | 447 (79.1)  368 (78.0)  24 (5.1)  15 (3.2)  5 (1.0)  9 (1.9)  33 (7.0)  4 (0.8)  6 (1.3)  8 (1.7) | 26 (28.3)  0  5 (18.5))  0  4 (14.8)  4 (14.8)  8 (29.6)  1 (3.7)  2 (7.4)  3 (11.1) | 473 (72.0)  368 (56.0)  29 (5.9)  15 (3.1)  9 (1.8)  13 (2.6)  41 (8.4)  5 (1.0)  8 (1.6)  3 (0.6) | < 0.001 |
| Preceding Illness: Yes, n (%) | 113 (20.0) | 31 (33.7) | 144 (21.9) | 0.005 |
| Primary ICD 10, n (%)  -Congenital  -Infection  -Endocrine  -Haematology/Oncology  -Endocrine  -Neuro/developmental/behavioural/psychiatric  -Cardiovascular  -Respiratory  -ENT  -Skin  -Kidney  -Conditions related to birth/prematurity  -Other | 0  105 (85.4)  0  0  0  0  7 (5.7)  0  1(0.8  1 (0.8)  0  1 (0.8)  8 (6.5) | 0  30(88.2)  0  0  1 (2.9)  0  2 (5.9)  0  0  0  0  0  1 (2.9) | 0  135 (86.0)  0  0  1 (0.6)  0  9 (5.7)  0  1 (0.6)  1 (0.6)  0  1 (0.6)  9 (5.7) |  |
| Chronic Illness: Yes, n (%)  Primary ICD 10, n (%) *  -Congenital  -Infection  - Haematology/Oncology  -Endocrine  -Neuro/developmental/behavioural/psychiatric  -Endocrine  -Cardiovascular  -Respiratory  -ENT  -Skin  -Kidney  -Conditions related to birth/prematurity  -Other | 510 (90.3)  169 (17.3)  2 (0.2)  17 (1.7)  9 (0.9)  29 (3.0)  608 (62.1)  20 (2.0)  9 (0.9)  30 (3.1)  2 (0.2)  10 (1.0)  53 (5.4)  21 (2.1) | 58 (63.0)  22 (22.9)  0  3 (3.1)  2 (2.1)  17 (17.7)  14 (14.6)  3 (3.1)  5 (5.2)  8 (8.3)  0  1 (1.0)  12 (12.5)  9 (9.3) | 568 (86.4)  191 (17.8)  2 (0.2)  20 (1.9)  11 (1.0)  46 (4.3)  622 (57.9)  23 (2.1)  14 (1.3)  38 (3.5)  2 (0.2)  11 (1.0)  65 (6.0)  30 (2.8) | <0.001 |
| Immunosuppression: Yes, n (%)  Missing, n | 22 (4.0)  12 | 6 (6.5)  0 | 28 (4.3)  12 | 0.269 |
| Fever: Yes, n (%)  Missing, n | 41 (7.6)  23 | 54 (58.7)  0 | 95 (15.0)  23 | < 0.001 |
| Long term antibiotics: Yes, n (%)  Missing, n | 31 (5.6)  9 | 10 (11.1)  2 | 41 (6.2)  11 | 0.078 |
| Recent antibiotics: Yes, n (%)  Missing, n | 103 (19.1)  26 | 25 (29.1)  6 | 128 (20.5)  32 | 0.046 |
| Lactate (mmol/l)  Median (IQR) | n=539  1.2 (0.9-1.9) | n=83  1.4 (0.9-2.3) | n=622  1.2 (0.9-1.9) | 0.186 |
| White Cell Count *10^9^ /l:  Median (IQR) | N=448  10.4 (7.2-14.4) | N=70  13.7 (7.5-20.52) | N=518  10.6 (7.2-15.2) | 0.008 |
| Neutrophil Count *10^9^ /l  Median (IQR) | N=488  6.8 (4.5-10.8) | N=69  11.4 (4.8-17.0) | N=517  7.3 (4.5 -11.4) | <0.001 |

Abbreviations: ^+^ Surgery: an operative procedure conducted in an operating theatre for the diagnostic or therapeutic treatment of diseases. This includes but is not limited to cardiac, neurosurgery, general, orthopaedic, thoracic, abdominal, burns / plastic and ENT procedures. IQR: Interquartile range, **can be more than one surgery ^ There can me more than one ICD 10 code within each category. ^Ɨ^ Long-term antibiotics defined as > 28 days duration.

S 3 Table: Number of patients with positive bacterial sterile site cultures or PCR results corresponding to admission Serious Bacterial Infection.

|  | Blood Culture | Blood PCR | CSF Culture | CSF PCR | Pleural fluid | Pleural fluid PCR | VP Shunt |
| --- | --- | --- | --- | --- | --- | --- | --- |
| Coagulase negative *Staphylococcus* | 1 |  |  |  |  |  | 1 |
| *Escherichia coli* | 2 |  |  |  |  |  |  |
| *Enterococcus faecalis* | 2 |  |  |  |  |  |  |
| Enterococcus spp. | 1 |  |  |  |  |  |  |
| *Klebsiella oxytoca* | 1 |  |  |  |  |  |  |
| *Lactococcus lactis* | 1 |  |  |  |  |  |  |
| *Moraxella catarrhalis* | 1 |  |  |  |  |  |  |
| *Neisseria meningitidis** | 4 | 8 |  | 2 |  |  |  |
| *Serratia liquefasiens* | 1 |  |  |  |  |  |  |
| *Streptococcus pneumoniae*** | 2 |  | 3 | 1 |  | 1 |  |
| *Streptococcus pyogenes* | 2 |  |  |  | 3 |  |  |
| Total | 18 | 8 | 3 | 3 | 3 | 1 | 1 |

In addition, two patients also had positive ASOT levels of 300 and 600

**Neisseria meningitidis:* 4 patients were blood culture and PCR positive (serogroup B x3, 1 serogroup W135), 8 cases blood PCR positive (7 serogroup B), 2 cases of CSF PCR positive serogroup B.

***Streptococcus pneumoniae:* 1 patient’s CSF & blood cultures positive, 1 positive blood culture, 2 CSF culture positive alone and 1 patient blood culture, CSF culture & CSF PCR positive.

**S 4 Table: Admission diagnosis of patients with Serious Bacterial Infection.**

| Clinical Diagnosis | Number of patients |
| --- | --- |
| Primary Bacteraemia | 13 |
| Central line associated bacteraemia | 5 |
| Empyema | 2 |
| Empyema & Bacteraemia | 2 |
| Pneumonia | 39 |
| Pneumonia & Bacteraemia | 1 |
| Ventilator Associated Pneumonia | 1 |
| Intra-abdominal infection | 7 |
| Meningitis | 3 |
| Meningitis & Bacteraemia | 4 |
| Necrotising enterocolitis | 2 |
| Other | 13 |
| Total | **92** |

S 5 Table: Comparison of baseline biomarker measurements for patients with and without Serious Bacterial Infection on admission

|  | SBI at Admission | | Median Difference, (95% CI for location), p-value |
| --- | --- | --- | --- |
|  | No  *N=565 (86%)* | Yes  *N=92 (14%)* |  |
| PCT  -Median (IQR) | N=544  0.1 (0.0 – 0.9) | N=85  7.5 (1.4 – 49.8) | < 0.001  5.8 (4.0 – 9.4) |
| Plasma NGAL  -Median (IQR) | N=236  116.7 (69.7 – 195.0) | N=59  268.1 (113.1-1000.0) | < 0.001  95.0 (42.9, 167.5) |
| Resistin  -Median (IQR) | N=218  40.6 (22.9 – 74.7) | N=55  133.8 (58.2 – 241.5) | < 0.001  85.2 (55.6 – 119.3) |
| APTT Slope  -Median (IQR) | N=470  -0.004 (-0.021-0.00) | N=81  -0.018 (-0.81—0.001) | <0.001  -0.018 (-0.028- -0.010) |
| APTT TR18  -Median (IQR) | N=470  99.8 (99.5 – 100.0) | N=81  99.4 (98.3 – 99.) | <0.001  -0.333 (-0.496- -0.194) |
| CRP  -Median (IQR) | N=446  0.0 (0.0 – 4.4) | N=74  58.4 (26.3 – 155.0) | <0.001  56.9 (46.3 -71.1) |

| Biomarkers and Cut points | Sensitivity (95% CI) | Specificity (95% CI) | PPV  (95% CI) | NPV  (95% CI) | Positive LR  (95% CI) | Negative LR (95% CI) | Model performance AUC (95% CI) |
| --- | --- | --- | --- | --- | --- | --- | --- |
| PCT > 5,  CRP >4 | 95.1  (89.7, 100) | 74.6  (70.5, 78.8) | 35.6  (23.6, 47.6) | 99.04  (98.1, 99.98) | 3.74  (3.14, 4.47) | 0.96  (0.02, 0.20) | 90.44  (86.76, 94.12) |
| PCT > 1.25,  CRP > 25 | 91.8  (84.9, 98.7) | 76.1  (72.0, 80.2) | 36.1  (24.1, 48.2) | 98.44  (97.3, 99.6) | 3.84  (3.18, 4.63) | 0.11,  (0.05, 0.25) | 90.80  (86.47, 95.12) |
| PCT > 2,  CRP >10,  APTT TR18 > 99.4,  APTT Slope -0.03 | 90.9  (83.3, 98.5) | 65.8  (60.8, 70.8) | 29.7  (17.7, 41.9) | 97.8  (96.3, 99.4) | 2.65  (2.25, 3.15) | 0.14  (0.06, 0.32) | 90.11  (85.14, 95.08) |
| PCT >2,  CRP > 10,  plasma NGAL 195,  APTT TR18 > 99.4,  APTT slope -0.03 | 91.9  (83.1, 100.) | 39.7  (31.7, 48.0) | 29.1  (14.4, 43.7) | 94.8  (91.1, 98.5) | 1.53  (1.29, 1.80) | 0.20  (0.07, 0.61) | 87.56 (80.27, 94.86) |
| PCT > 2,  CRP > 10,  plasma NGAL > 195,  APTT TR 18 > 99.4,  APTT slope > -0.03  resistin > 96.28 | 97.6  (91.4, 100) | 39.7  (31.1, 48.2) | 30.9  (14.8, 45.7) | 98.0  (95.6, 100) | 1.61  (1.38, 1.88) | 0.07  (0.01, 0.52) | 91.14  (85.63, 96.66) |

**S 6 Table: Combinations of biomarkers based upon pre-specified cut-points and performance.**

S 7 Table: Independent variables statistically significantly associated with the dichotomous outcome prolonged PICU stay on univariate analysis.

Data presented as means +/- SE, median and range or number (%) for binary/categorical variables.

|  | PICU Stay: Below Median (n=328; 49.92%) | PICU Stay: Above Median (n=329; 50.08%) | Missing values | P value |
| --- | --- | --- | --- | --- |
| Age | 3.92+/-0.27;  1.29 (0-16.43) | 2.98+/-0.24;  0.77 (0-16.23) | 0 | <0.005 |
| Surgery  -Cardiac  -None  -Other | 244 (60.9%)  58 (31.5%)  46 (43.8%) | 144 (39.1%)  126 (68.5%)  59 (56.2%) | 0 | <0.005 |
| Bypass time | 101.23+/-4;  97 (0-317) | 129.32+/-6.45;  129 (0-360) | 295 | <0.005 |
| Cross clamp time | 61.03+/-3.22;  56 (0-222) | 80.57+/-4.89;  77 (0-231) | 299 |  |
| Recruitment to CHIP:  No  Yes | 317 (51.5%)  8 (21.1%) | 298 (48.5%)  30 (78.9%) | 4 | <0.005 |
| Type of admission:  -Planned  -Unplanned (emergency)  -Unplanned following surgery | 237 (62.2%)  79 (31.1%)  12 (54.5%) | 144 (37.8%)  175 (68.9%)  10 (45.5%) | 0 | <0.005 |
| SBI at admission  -No  -Yes | 301 (53.3%)  27 (29.3%) | 264 (46.7%)  65 (70.7%) | 0 | <0.005 |
| Inotrope score (in first 12 hours) | 5.95+/-0.43;  5 (0-80) | 15.28+/-1.69;  6.66 (0-355) | 2 | <0.005 |
| Fever  -No  -Yes | 285 (52.9%)  28 (29.5%) | 254 (47.1%)  67 (70.5%) | 23 | <0.005 |
| Long term antibiotics  -No  -Yes | 309 (51.1%)  13 (31.7%) | 296 (48.9%)  28 (68.3%) | 11 | 0.03 |
| Recent antibiotics  -No  -Yes | 268 (53.9%)  45 (35.2%) | 229 (46.1%)  83 (64.8%) | 32 | <0.005 |
| Antibiotics prior to ward  -No  -Yes | 257 (53%)  57 (40.4%) | 228 (47%)  84 (59.6%) | 31 | 0.01 |
| General appearance  -No comment  -Unwell  -Well | 63 (48.8%)  40 (33.1%)  154 (61.8%) | 66 (51.2%)  81 (66.9%)  95 (38.2%) | 158 | <0.005 |
| PCT | 4.4+/-1.55;  0.11 (0-424) | 21.76+/-5.83;  0.6 (0-1134) | 28 | <0.005 |
| PIM | 0.04+/-0;  0.02 (0-0.33) | 0.09+/-0.01;  0.05 (0.01-0.76) | 445 | <0.005 |
| PIM2 | 0.03+/-0;  0.02 (0-0.2) | 0.07+/-0.01;  0.05 (0.01-0.54) | 445 | <0.005 |
| Platelet | 197.12+/-6.5;  169 (14-507) | 229.39+/-8.6;  213 (16-938) | 139 | 0.01 |
| CRP | 15.69+/-2.8;  0 (0-303.7) | 31.94+/-3.98;  0 (0-401.7) | 137 | < 0.005 |
| Lactate | 31.94+/-3.98;  0 (0-401.7) | 2.01+/-0.11;  1.39 (0.02-17.06) | 35 | < 0.005 |
| PELOD day 1 | 9.26+/-0.29;  11 (0-23) | 12.41+/-0.36;  12 (0-43) | 3 | < 0.005 |

S 8 Table: Final model of best fit for prolonged PICU stay.

|  | **Coefficient** | **Standard Error** | **Odds Ratio** | **95% CI** | **P value** |
| --- | --- | --- | --- | --- | --- |
| Intercept | -2.64 | 0.40 | 0.07 | 0.03-0.15 | <0.001 |
| Admission type: Unplanned (emergency) | 1.73 | 0.30 | 5.62 | 3.20-10.19 | <0.001 |
| Admission type: Unplanned following surgery | 2.11 | 0.67 | 8.25 | 2.32- 34.11 | <0.001 |
| Inotrope Score in 1^st^ 12 hours | 0.04 | 0.01 | 1.04 | 1.01 – 1.07 | 0.002 |
| Age | -0.092 | 0.03 | 0.91 | 0.86-0.96 | <0.001 |
| Recruitment to CHiP | 1.23 | 0.47 | 3.43 | 1.41-9.01 | 0.008 |
| Long Term Antibiotics:  Yes | 1.58 | 0.57 | 4.89 | 1.66-16.98 | 0.005 |
| PELOD | 0.07 | 0.02 | 1.07 | 1.03-1.12 | <0.001 |
| Platelet | 0.002 | 0.001 | 1.00 | 1.007 -1.004 | 0.009 |
| Lactate | 0.26 | 0.10 | 1.30 | 1.08-1.60 | 0.010 |
| Antibiotics Prior to Ward: Yes | -0.63 | 0.32 | 0.53 | 0.28-0.99 | 0.049 |

Supplementary Figure 1. ROC Curve for Prolonged PICU Stay


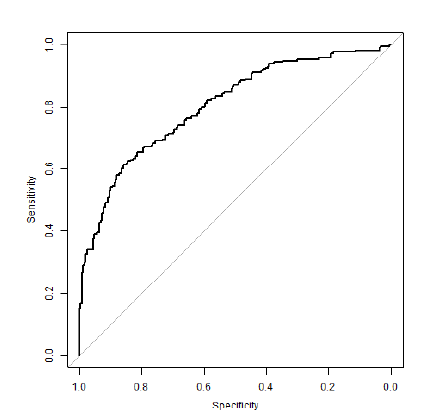


*AUC 80.2% 95% CI: 0.7628-0.8416

Supplementary Table 9: Independent variables statistically significantly associated with the continuous variable duration of PICU stay (at 5% level).

Data presented as means +/- SE, median and range or number (%) for binary/categorical variables.

|  | Summary Statistics | PICU days summary statistics | Missing values | P value |
| --- | --- | --- | --- | --- |
| Age | 3.45+/-0.18; 1.01 (0-16.43) | 6.61+/-0.4; 3.29  (0.25-127.12) | 0 | <0.005 |
| Surgery  -Cardiac  -None  -Other | 368 (56%)  184 (28%)  105 (16%) | 5.35+/-0.5; 2.74 (0.25-127.12)  8.02+/-0.69;4.82 (0.45-57.21)  8.54+/-1.25; 3.95 (0.53-78.07) | 0 | <0.005 |
| Bypass time | 112.33+/-3.58; 108.5 (0-360) | 6.61+/-0.4; 3.29 (0.25-127.12) | 295 | <0.005 |
| Cross clamp time | 68.73+/-2.78; 65 (0-231) | 6.61+/-0.4;3.29 (0.25-127.12) | 299 | <0.005 |
| Recruitment to CHiP:  No  Yes | 615 (94.2%)  38 (5.8%) | 6.5+/-0.42; 3.17 (0.25-127.12)  8.64+/-1.36; 6.92 (0.54-45.57) | 4 | <0.005 |
| Type of admission:  -Planned  -Unplanned (emergency)  -Unplanned following surgery | 381 (58%)  254 (38.7%)  22 (3.3%) | 5.47+/-0.54; 2.42 (0.25-127.12)  8.45+/-0.61; 5.02 (0.45-57.21)  5.06+/-1.2; 3.22 (0.53-24.71) | 0 | <0.005 |
| SBI at admission  -No  -Yes | 565 (86%)  92 (14%) | 6.35+/-0.43; 2.95 (0.25-127.12)  8.17+/-0.97; 5.42 (0.53-45.95) | 0 | <0.005 |
| Inotrope score (in first 12 hours) | 10.61+/-0.89; 5 (0-355) | 6.61+/-0.4; 3.29 (0.25-127.12) | 2 | <0.005 |
| Fever  -No  -Yes | 539 (85%)  95 (15%) | 6.39+/-0.45; 3.02 (0.25-127.12)  8.23+/-0.93; 5.06 (1.41-45.95) | 23 | <0.005 |
| Long term antibiotics  -No  -Yes | 605 (93.7%)  41 (6.3%) | 6.24+/-0.39; 3.19 (0.45-127.12)  11.53+/-2.47; 6.28 (0.25-76.22) | 11 | <0.005 |
| Recent antibiotics  -No  -Yes | 497 (79.5%)  128 (20.5%) | 6.15+/-0.47; 2.93 (0.25-127.12)  7.4+/-0.68; 5.15 (0.53-57.21) | 32 | <0.005 |
| Antibiotics prior to ward  -No  -Yes | 485 (77.5%)  141 (22.5%) | 6.26+/-0.47; 3.01 (0.25-127.12)  7.31+/-0.76; 4.48 (0.48-52.97) | 31 | <0.005 |
| General appearance  -No comment  -Unwell  -Well | 129 (25.9%)  121 (24.2%)  249 (49.9%) | 7.31+/-0.76;4.48 (0.48-52.97)  6.91+/-0.69; 4.5 (0.53-45.79)  4.86+/-0.43; 2.61 (0.25-57.21) | 158 | <0.005 |
| PCT | 12.96+/-3; 0.22 (0-1134) | 6.61+/-0.4; 3.29 (0.25-127.12) | 28 | <0.005 |
| Plasma NGAL | 255.18+/-17.81; 125.32 (0-1000) | 6.61+/-0.4; 3.29 (0.25-127.12) | 362 | 0.020 |
| PIM | 0.06+/-0.01; 0.03 (0-0.76) | 6.61+/-0.4;3.29 (0.25-127.12) | 445 | <0.005 |
| PIM2 | 0.05+/-0; 0.03 (0-0.54) | 6.61+/-0.4;3.29 (0.25-127.12) | 445 | <0.005 |
| Platelet | 212.38+/-5.36; 187.5 (14-938) | 6.61+/-0.4;3.29 (0.25-127.12) | 139 | 0.020 |
| CRP | 23.28+/-2.41; 0 (0-401.7) | 6.61+/-0.4; 3.29 (0.25-127.12) | 137 | < 0.005 |
| Lactate | 1.71+/-0.06; 1.25 (0.02-17.06) | 6.61+/-0.4; 3.29 (0.25-127.12) | 35 | < 0.005 |
| PELOD day 1 | 10.83+/-0.24; 11 (0-43) | 6.61+/-0.4; 3.29 (0.25-127.12) | 3 | < 0.005 |

Supplementary Table 10: Final Model of Best fit for Log (Length of PICU Stay).

|  | Coefficient | Standard error | Expo (Coef) | 95% CI | P value |
| --- | --- | --- | --- | --- | --- |
| Intercept | **0.33** | **0.15** | **1.39** | **1.03-1.86** | **0.03** |
| Age | -0.04 | 0.01 | 0.96 | 0.95-0.98 | <0.01 |
| Surgery: None | 0.63 | 0.21 | 1.88 | 1.23-2.86 | <0.01 |
| Surgery: Other | 0.78 | 0.19 | 2.18 | 1.50-3.16 | <0.01 |
| Recruitment to CHiP | 0.48 | 0.16 | 1.61 | 1.16 -2.22 | <0.01 |
| Bypass Time | 0.00 | 0.00 | 1.00 | 1.001-1.04 | <0.01 |
| Type of Admission: Unplanned (emergency) | 0.40 | 0.18 | 1.50 | 1.04-2.15 | 0.03 |
| Type of Admission: Unplanned following surgery | 0.02 | 0.25 | 1.02 | 0.62 - 1.69 | 0.92 |
| Inotropes | 0.01 | 0.00 | 1.01 | 1.003-1.01 | <0.01 |
| Long Term Antibiotics: Yes | 0.42 | 0.18 | 1.53 | 1.07-2.18 | 0.02 |
| Recent Antibiotics: Yes | 0.29 | 0.11 | 1.33 | 1.08-1.65 | 0.01 |
| Antibiotics prior to ward: Yes | -0.25 | 0.11 | 0.78 | 0.62-0.97 | 0.03 |
| General appearance: unwell | -0.16 | 0.12 | 0.86 | 0.67-1.08 | 0.19 |
| General appearance: well | -0.05 | 0.11 | 0.95 | 0.78-1.17 | 0.65 |
| PCT | -0.01 | 0.00 | 1.00 | 0.998-1.001 | 0.18 |
| PELOD | 0.03 | 0.01 | 1.03 | 1.02-1.04 | <0.01 |

Coefficient of determination (R^2^ ) for model 25.9%.

Supplementary Table 11: Independent variables statistically significantly associated with number of ventilator days (at 5% level).

Data presented as means +/- SE, median and range or number (%) for binary/categorical variables.

|  | Summary Statistic | Ventilator days summary statistics | Missing values | P value |
| --- | --- | --- | --- | --- |
| Age | 3.45+/-0.18; 1.01 (0-16.43) | 4.38 +/- 0.43; 1.92 (0.07-189.97) | 0 | < 0.005 |
| Surgery  -Cardiac  -None  -Other | 368 (56%)  184 (28%)  105 (16%) | 3.73 +/- 0.65; 1.17 (0.1-189.97)  5.38+/-0.5; 3.25 (0.36-35.49)  5.3+/-0.89; 2.58 (0.07-66.08) | 0 | <0.005 |
| Bypass time | 112.33+/-3.58; 108.5 (0-360) | 4.38+/-0.43; 1.92 (0.07-189.97) | 295 | <0.005 |
| Cross clamp time | 68.73+/-2.78; 65 (0-231) | 4.38+/-0.43; 1.92 (0.07-189.97) | 299 | < 0.005 |
| Chronic illness  -No  -Yes | 88 (13.4%)  569 (86.6%) | 4.04+/-0.58; 2.66 (0.09-35.49)  4.43+/-0.49; 1.77 (0.07-189.97) | 0 | 0.030 |
| Recruitment to CHiP:  No  Yes | 615 (94.2%)  38 (5.8%) | 4.32+/-0.46; 1.77 (0.07-189.97)  5.74+/-0.77; 5.23 (0.62-24) | 0 | < 0.005 |
| Type of admission:  -Planned  -Unplanned (emergency)  -Unplanned following surgery | 381 (58%)  254 (38.7%)  22 (3.3%) | 2.92+/-0.38; 1.14 (0.07-120.56)  6.92+/-0.99; 3.69 (0.22-189.97)  3.62+/-1.28; 1.79 (0.19-24.13) | 0 | < 0.05 |
| SBI at admission  -No  -Yes | 565 (86%)  92 (14%) | 4.25+/-0.49; 1.68 (0.07-189.97)  5.27+/-0.63; 3.79 (0.4-35.49) | 0 | < 0.005 |
| Inotrope score (in first 12 hours) | 10.61+/-0.89; 5 (0-355) | 4.38+/-0.43; 1.92 (0.07-189.97) | 2 | < 0.005 |
| Fever  -No  -Yes | 539 (85%)  95 (15%) | 4.24+/-0.5; 1.65 (0.07-189.97)  5.69+/-0.67; 3.81 (0.1-35.49) | 23 | < 0.005 |
| Long term antibiotics  -No  -Yes | 605 (93.7%)  41 (6.3%) | 3.98+/-0.31; 1.89 (0.07-120.56)  12.78+/-6.94; 4 (0.1-189.97) | 11 | 0.020 |
| Recent antibiotics  -No  -Yes | 497 (79.5%)  128 (20.5%) | 3.68+/-0.33; 1.69 (0.07-120.56)  5.24 +/-0.77; 3.22 (0.1-66.08) | 32 | <0.005 |
| Antibiotics prior to A&E  -No  -Yes | 603 (96.2%)  24 (3.8%) | 3.96+/-0.31; 1.88 (0.07-120.56)  5.99+/-1.41; 4.19 (0.4-23.83) | 30 | 0.030 |
| Antibiotics prior to ward  -No  -Yes | 485 (77.5%)  141 (22.5%) | 3.81+/-0.36; 1.6 (0.07-120.56)  4.82+/-0.5; 3.23 (0.15-35.49) | 31 | <0.005 |
| General appearance  -No comment  -Unwell  -Well | 129 (25.9%)  121 (24.2%)  249 (49.9%) | 4.1+/-0.51; 2.13 (0.12-31.27)  4.33+/-0.38; 3.23 (0.07-20.35)  2.9+/-0.3; 1.24 (0.1-35.49) | 158 | < 0.005 |
| PCT | 12.96+/-3; 0.22 (0-1134) | 4.38+/-0.43; 1.92 (0.07-189.97) | 28 | < 0.005 |
| Plasma NGAL | 255.18+/-17.81; 125.32 (0-1000) | 4.38+/-0.43; 1.92 (0.07-189.97) | 362 | 0.010 |
| Resistin | 128.67+/-17.93; 48.86 (0-1500) | 4.38+/-0.43; 1.92 (0.07-189.97) | 384 | 0.040 |
| PIM* | 0.06+/-0.01; 0.03 (0-0.76) | 4.38+/-0.43; 1.92 (0.07-189.97) | 445 | <0.005 |
| PIM2 | 0.05+/-0; 0.03 (0-0.54) | 4.38+/-0.43; 1.92 (0.07-189.97) | 445 | <0.005 |
| Platelet | 212.38+/-5.36; 187.5 (14-938) | 4.38+/-0.43; 1.92 (0.07-189.97) | 139 | <0.05 |
| CRP | 23.28+/-2.41; 0 (0-401.7) | 4.38+/-0.43; 1.92 (0.07-189.97) | 137 | <0.005 |
| Base excess | -2.1+/-0.2; -2.4 (-25.4-26.7) | 4.38+/-0.43; 1.92 (0.07-189.97) | 31 | 0.030 |
| Lactate | 1.71+/-0.06; 1.25 (0.02-17.06 | 4.38+/-0.43; 1.92 (0.07-189.97) | 35 | <0.005 |
| PELOD day 1 | 10.83+/-0.24; 11 (0-43) | 4.38+/-0.43; 1.92 (0.07-189.97) | 3 | <0.005 |

*Paediatric Index of Mortality Score (PIM) [38] [39]

|  | **Coefficient** | **Standard Error** | **Expo (Coef)** | **95% CI** | **p-value** |
| --- | --- | --- | --- | --- | --- |
| (Intercept) | -1.09 | 0.23 | 0.34 | 0.21-0.53 | <0.001 |
| Age | -0.07 | 0.01 | 0.93 | 0.90-0.85 | <0.001 |
| Surgery: None | 0.48 | 0.32 | 1.62 | 0.87-3.05 | 0.134 |
| Surgery: Other | 0.77 | 0.30 | 2.15 | 1.19-3.88 | 0.012 |
| Bypass Time | 0.00 | 0.00 | 1.00 | 1.002-1.01 | <0.001 |
| Type Admission: Unplanned (emergency) | 1.18 | 0.28 | 3.24 | 1.86-6.66 | <0.001 |
| Type Admission: Unplanned following surgery | 1.08 | 0.37 | 2.93 | 1.40-6.13 | 0.004 |
| Recruitment to CHiP: Yes | 0.87 | 0.20 | 2.38 | 1.60- 3.56 | <0.001 |
| BI Admission: Yes | -0.45 | 0.21 | 0.64 | 0.42-0.96 | 0.03 |
| Inotrope | 0.01 | 0.00 | 1.01 | 1.001-1.011 | 0.022 |
| Long Term antibiotics: Yes | 0.62 | 0.31 | 1.85 | 0.996-3.44 | 0.051 |
| Recent Antibiotics: Yes | 0.22 | 0.15 | 1.25 | 0.93-1.68 | 0.14 |
| General Appearance: unwell | -0.29 | 0.16 | 0.75 | 0.54-1.03 | 0.08 |
| General Appearance: well | -0.02 | 0.14 | 0.98 | 0.73-1.30 | 0.87 |
| Platelet | 0.00 | 0.00 | 1.00 | 0.99-1.02 | 0.13 |
| CRP | 0.00 | 0.00 | 1.00 | 1-1.01 | 0.05 |
| BE | 0.03 | 0.01 | 1.03 | 1.01-1.10 | 0.02 |
| Lactate | 0.09 | 0.04 | 1.09 | 1.01-1.19 | 0.04 |
| PELOD | 0.04 | 0.01 | 1.04 | 1.02-1.07 | <0.001 |

**Supplementary Table 12: Final model of best fit for log (duration of ventilation)**

Coefficient of determination (R^2^ ) 45.0%.

**Supplementary Figure 2. ROC curve for the final model using the first recorded values of blood biomarker data for prolonged PICU Stay**.

* AUC 68.05%, 95% CI 63.93% to 72.16%

Supplementary Figure 3. ROC curve of the final model using the maximum values of the blood biomarker data for prolonged PICU Stay.


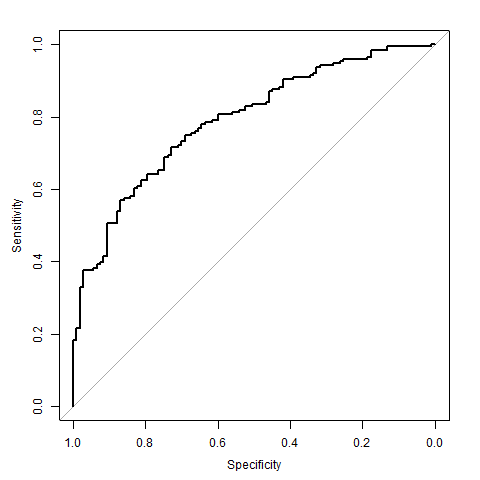


* AUC 78.95%, 95% CI 73.73% to 84.16%
