# Supplementary material for: Procalcitonin, C-reactive protein, neutrophil gelatinase-associated lipocalin, resistin and the APTT waveform for the early diagnosis of serious bacterial infection and prediction of outcome in critically ill children
Source: PLoS One. 2021 Feb 5;16(2):e0246027. doi: 10.1371/journal.pone.0246027 (PMC7864456; doi:10.1371/journal.pone.0246027)
Supplement: Attachment — Submitted filename: Response to Reviewers v 3.docx [file pone.0246027.s002.docx]

Clinical Infection, Microbiology, and Immunity

Institute of Veterinary and Ecological Science

University of Liverpool

8 West Derby Street

Liverpool

L7 3AE

2^nd^ December 2020

Dear Editor

Thank you for completing initial checks on our Manuscript entitled “Procalcitonin, C-Reactive Protein, Neutrophil Gelatinase-Associated Lipocalin, Resistin and the APTT Waveform for the early diagnosis of serious bacterial infection and prediction of outcome in critically ill children”. In response to the issues highlighted we have made the following changes to the manuscript.

- **Ethics Statement –** We have removed the ethics statement previously present on page 22 of the manuscript. We have moved the ethics statement that was already present under the “Participants” sub-head of the methods section to a separate sub-heading (Ethical Approval and Sample Size Calculation) to ensure this is more easily visible to readers.
- **Data Availability Statement –** The data that support the findings of this study are available on request from the University of Liverpool, Institute of Infection, Veterinary and Ecological Sciences Head of Operations [iveshoo@liverpool.ac.uk](mailto:iveshoo@liverpool.ac.uk). The data are not publicly available due to restrictions imposed by collection of patient data from a named hospital, and therefore contains information that could compromise the privacy of research participants. The data sharing statement on p22 of the manuscript has been amended accordingly.
- **Competing Interests Statement –** We can confirm that the suggested competing interest statement proposed by the journal is correct. We have updated this on p23 of the manuscript.

These changes have been uploaded to the submission portal as “Manuscript v 3” and “Revised Manuscript with tracked changes v3”.

Please do not hesitate to contact me if you require any further information.

Yours sincerely

Dr Maryke Nielsen

Corresponding Author
